# Human cytomegalovirus gH stability and trafficking are regulated by ER-associated degradation and transmembrane architecture

**DOI:** 10.1038/srep23692

**Published:** 2016-03-30

**Authors:** Thomas J. Gardner, Rosmel E. Hernandez, Vanessa M. Noriega, Domenico Tortorella

**Affiliations:** 1 Icahn School of Medicine at Mount Sinai, Department of Microbiology, New York, NY 10029, USA

## Abstract

The prototypic betaherpesvirus human cytomegalovirus (CMV) establishes life-long persistence within its human host. While benign in healthy individuals, CMV poses a significant threat to the immune compromised, including transplant recipients and neonates. The CMV glycoprotein complex gH/gL/gO mediates infection of fibroblasts, and together with the gH/gL/UL128/130/131 a pentameric complex permits infection of epithelial, endothethial, and myeloid cells. Given the central role of the gH/gL complex during infection, we were interested in studying cellular trafficking of the gH/gL complex through generation of human cells that stably express gH and gL. When expressed alone, CMV gH and gL were degraded through the ER-associated degradation (ERAD) pathway. However, co-expression of these proteins stabilized the polypeptides and enhanced their cell-surface expression. To further define regulatory factors involved in gH/gL trafficking, a CMV gH chimera in which the gH transmembrane and cytoplasmic tail were replaced with that of human CD4 protein permitted cell surface gH expression in absence of gL. We thus demonstrate the ability of distinct cellular processes to regulate the trafficking of viral glycoproteins. Collectively, the data provide insight into the processing and trafficking requirements of CMV envelope protein complexes and provide an example of the co-opting of cellular processes by CMV.

Human cytomegalovirus (CMV) is a member of the β-herpesvirus subfamily, which has a seroprevalence of 60–90% in adults worldwide^1^. While normally asymptomatic, the virus can cause morbidity and mortality in susceptible individuals, including transplant recipients and neonates who are infected when the virus crosses the placental barrier during embryonic development. In these patient populations CMV infection poses significant health risks, causing the US Institute of Medicine to declare the development of a CMV vaccine a priority^2^. With the aim of developing effective vaccine strategies, recent research has focused intensely on the development of potent neutralizing antibodies that target the glycoprotein complexes on the surface of the CMV virion which are crucial for cell attachment, binding, and fusion^3^. A thorough understanding of the expression and processing of the various CMV glycoprotein complexes should thus prove critical in facilitating the development of therapeutics targeting virus-infected cells.

All herpesviruses utilize the conserved core fusion machinery that consists of gB and the gH/gL heterodimer. During CMV infection, gH and gL complex with additional viral proteins in the ER, including gO and UL128, UL130, and UL131a. In addition to increasing the ER export of gH/gL^4^,^5^,^6^, the assembly of these complexes enables nascent viruses to infect their full range of cell targets. The formation of the gH/gL/gO complex versus the gH/gL/U128/130/131 a pentamer in the ER is critically important, as the complexes carry out cell-type dependent mechanisms of cell entry. Entry into fibroblasts occurs via fusion at the cell surface through the gH/gL/gO complex^7^, while entry into epithelial, endothelial, dendritic cells and monoctyes occurs through pH-dependent endoctytosis and requires both gH/gL/gO and the pentameric complex^6^,^7^,^8^,^9^,^10^,^11^,^12^.

Recent reports have shed light on the complex protein regulation that occurs in the ER that permits gH/gL heterodimers to assemble into fusion-competent glycoprotein complexes. This includes the identification of a single cysteine residue in gL that forms an exclusive disulfide bond with either gO or UL128^13^, as well as the discovery of an ER-resident CMV protein, UL148, which may regulate the ratio of gH/gL/gO to PC and thus the subsequent tropism of nascent virions by competing with UL128 for binding to the gH/gL dimer^14^. These studies illustrate the tightly regulated viral processing events that ensure proper assembly of CMV glycoprotein complexes during infection and indicate their paramount importance in preserving viral fitness.

While recent work has described the regulation involved in assembling mature gH/gL-containing complexes, we sought to understand the nature of gH/gL dimerization and its effects on gH stability and processing. Thus, to delineate the processes involved in gH trafficking, we engineered U373 astrocytoma cell lines that stably express gH and gL proteins. Biochemical and cell fluorescent analysis revealed that co-expression of gL stabilizes gH by limiting its degradation by the proteasome, and permitting its cell-surface expression. Dimerization with gL was not an absolute requirement for ER escape by gH however, as the gH transmembrane domain and cytosolic tail were found to play a regulatory role to enhance gH trafficking to the cell surface. These findings demonstrate that the stability and trafficking of gH can be regulated through distinct processes, which likely serve critical roles in quality control of the viral glycoprotein complexes generated during CMV infection.

## Results

### CMV envelope gH protein is sequestered within the cell during a CMV infection

**Figure 1:**
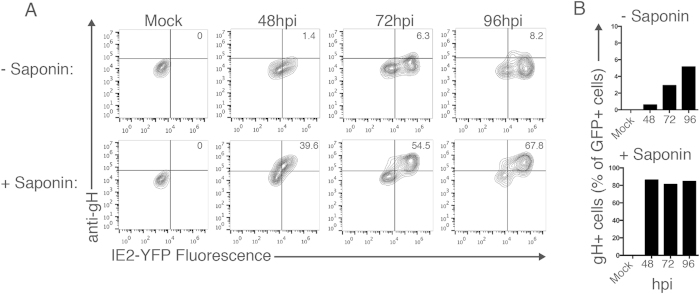
gH is localized within the cell during a CMV infection. (**A**) MRC5 fibroblasts infected with the CMV reporter strain AD169_IE2-YFP_ (MOI: 5) were harvested between 48 and 96 hpi and analyzed by flow cytometry for YFP expression (x-axis) and gH expression (y-axis). Cells were either untreated (top row) or permeabilized with saponin (bottom row) to distinguish between extracellular versus intracellular gH protein. Percent of double-positive (YFP+, gH+) cells is indicated in the top right panel. (**B**) Percent of YFP + cells that were positive for gH was determined for non permeabilized (top panel) and permeabilized (bottom panel) samples from all time points.

The gH/gL complexes are critical for the generation of infectious virions. Thus, we were interested in examining the kinetics of gH/gL surface expression during a virus infection. To analyze virus-infected cells, MRC5 fibroblast cells were infected (MOI: 5) with a reporter CMV virus strain that expresses a chimeric IE2 protein coupled to a Yellow Fluorescent Protein (YFP) (AD169_IE2-YFP_). Infection with the AD169_IE2-YFP_ strain permits infected cells to be distinguished from non-infected cells based on YFP fluorescence^15^,^16^. Although the AD169 genome lacks the ULb’ region, which encodes the ER-resident UL148 as well as the pentameric complex component UL131a, the virus forms gH/gL/gO complexes which are capable of infecting fibroblast cells, and provides a tool for the study of post translational regulation of gH/gL complexes^8^,^9^,^17^. We harvested cells throughout a 96-hour time course and subjected them to immunostaining with the anti-gH antibody 14-4b followed by flow cytometry analysis (Fig. 1A). To reveal the proportion of gH present on the surface of infected cells versus total intracellular gH, cells were either left untreated or permeabilized with saponin prior to antibody incubation. The efficacy of saponin treatment was confirmed through staining of the cytoskeletal protein α-tubulin in permeabilized U373 cells but not on intact cells to distinguish the labeling between intracellular and membrane surface proteins (Fig. S1). Flow cytometry analysis revealed the expression of gH only in infected (YFP + ) cells, beginning at 48 hpi and increasing through 96 hpi with only a fraction of the total amount of gH observed on the cell surface. Surface gH expression increased throughout the time course, peaking at 96 hpi with only ~8% of the cells positive for gH, while ~68% of cells were positive for gH following permeabilization. Analysis of YFP-positive cells revealed the low percentage of CMV-infected cells that express gH on the surface, while permeabilized cells maintained a high percentage of gH expression at all time points tested (Fig. 1B). The data indicate that gH expression on the cell surface represents a small fraction of the total amount of gH produced in a lytic CMV infection, and that post translational regulatory events may limit the amount of the gH/gL complex that traffics to the cell surface.

### CMV gH and gL proteins are stabilized upon their co-expression

**Figure 2:**
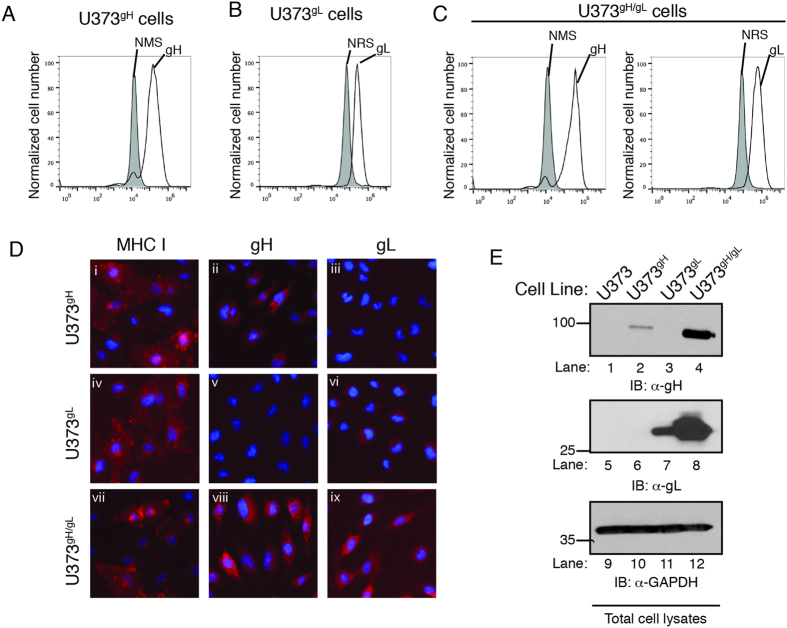
gH and gL are stabilized when co-expressed in the same cell. U373^gH^ (**A**), U373^gL^ (**B**) and U373^gH/gL^ (**C**) cells were permeabilized and analyzed by flow cytometry for intracellular expression of gH or gL. Background fluorescence levels were established by incubating the cells with normal mouse serum (NMS) or normal rabbit serum (NRS) (gray peaks). (**D**) U373^gH^, U373^gL^ and U373^gH/gL^ cells were subjected to analysis by fluorescent microscopy. Cells were probed for properly-folded MHC class I (i, iv, and vii), gH (ii, v, and viii), or gL (iii, vi, and ix) using the respective antibodies followed by incubation with an IgG species-specific Alexa-555 fluorescent antibody. Hoechst reagent allowed visualization of the nucleus. (**E**) Total cell lysates from U373, U373^gH^, U373^gL^, and U373^gH/gL^ were subjected to immunoblot analysis for gH (lanes 1–4), gL (lanes 5–8), and glyceraldehyde-3-phosphate dehydrogenase (GAPDH) (lanes 9–12). The relative molecular weight standards and polypeptides are indicated.

In order to study the cellular processes that contribute to trafficking of the CMV gH/gL envelope complex, we chose to express gH and gL in human cells in the absence of additional viral proteins. Human astrocytoma (U373) cell lines were stably transduced with retroviral vectors encoding gH or gL derived from the CMV TB40/E strain. Transduced cell pools were selected to generate pure populations of U373^gH^ and U373^gL^ cells. Finally, U373^gH/gL^ cell lines were generated by transducing U373^gH^ cells with gL to ensure that equivalent amounts of gH were expressed in both cell lines. The gH gene was delivered with a retroviral vector encoding green fluorescent protein (GFP), thus GFP fluorescence levels in U373^gH^ and U373^gH/gL^ cells was measured by flow cytometry to verify the equal gH transduction levels in the respective cell lines (Fig. S1). The gH and gL protein levels in saponin-treated U373^gH^, U373^gL^ , and U373^gH/gL^ cells were analyzed by intracellular flow cytometry with normal mouse or normal rabbit serum as controls (Fig. 2A–C, gray peak). Specific labeling for both gH and gL was observed in the respective cell line, validating their use to study the expression and trafficking of CMV gH and gL glycoproteins.

The localization of gH and gL proteins in U373^gH^, U373^gL^, and U373^gH/gL^ cells was analyzed by indirect fluorescent microscopy (Fig. 2D). Antibodies directed against properly folded MHC class I demonstrated appropriate experimental conditions (Fig. 2D, i, iv, and vii). gH was observed in U373^gH^ and U373^gH/gL^ cells, but not U373^gL^ cells. Similarly, gL was detected exclusively in U373^gL^ and U373^gH/gL^ cells. In U373^gH^ cells, gH appeared in a perinuclear expression pattern (Fig. 2D, ii) and gL in U373^gL^ cells displayed a similar perinuclear configuration (Fig. 2D, vi). Intriguingly, both gH and gL expression appeared to be enhanced in U373^gH/gL^ cells, with both proteins displaying an increased, and more diffuse fluorescent intensity extending beyond perinuclear regions and consistent with a Golgi network pattern (Fig. 2D, viii and ix).

We next examined protein expression from total cell lysates of U373, U373^gH^, U373^gL^ and U373^gH/gL^ cells by immunoblot analysis (Fig. 2E). CMV gH was exclusively expressed in U373^gH^ and U373^gH/gL^ cells (Fig. 2E, lanes 2 and 4) and gL was detected in U373^gL^ and U373^gH/gL^ cells only (Fig. 2E, lanes 7 and 8). Anti-GAPDH immunoblot confirmed equivalent protein loading (Fig. 2E, lanes 9–12). The levels of gH and gL were dramatically enhanced in U373^gH/gL^ cells compared to cells that expressed individual proteins (Fig. 2E, lanes 4 and 8). These results suggest that gH and gL enhance each others stability when expressed in the same cell, and are supported by previous findings indicating that herpes simplex virus 1 (HSV-1) glycoprotein gH forms a complex with gL, which influences the folding and surface expression of gH^11^.

### Non-complexed gH and gL are degraded in a proteasome-dependent manner

**Figure 3:**
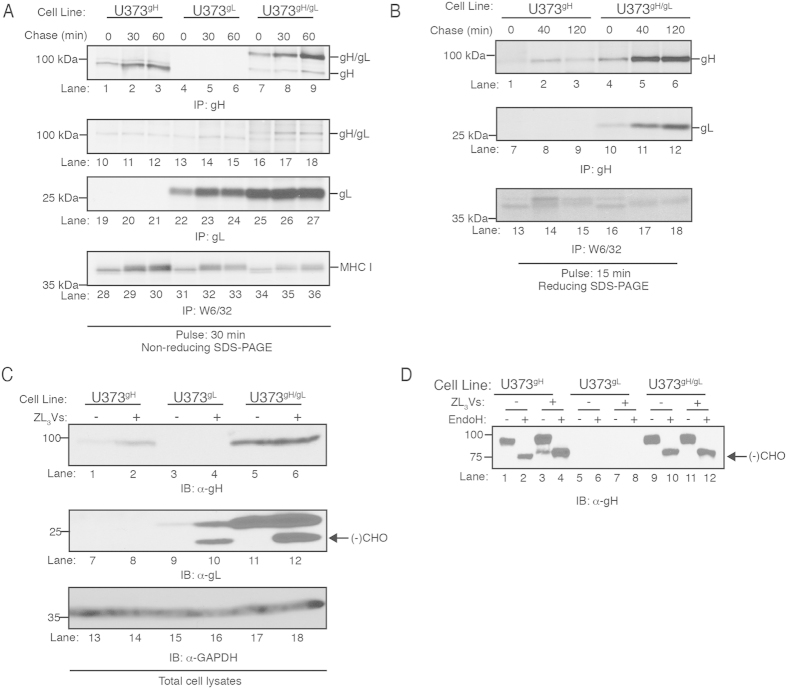
gH and gL form a heterodimer that is resistant to proteasome degradation. (**A**) U373^gH^, U373^gL^, and U373^gH/gL^ cell lines were metabolically labeled with ^35^S-methionine for 30 minutes and then chased for 60 minutes. Cell lysates harvested at 0, 30 and 60 minutes chase times were subjected to immunoprecipitation with gH (lanes 1–9), gL (10–18 and 19–27), and properly-folded MHC class I (lanes 28–36) antibodies, followed by resolution with non-reducing SDS-PAGE. (**B**) U373^gH^ and U373^gH/gL^ cell lines were metabolically labeled with ^35^S-methionine for 15 minutes and then chased for 120 minutes. Cell lysates harvested at 0, 40 and 120 minutes chase times were subjected to immunoprecipitation with gH (lanes 1–12) and properly-folded MHC class I (lanes 13–18) antibodies, followed by resolution with reducing SDS-PAGE. (**C**) U373^gH^, U373^gL^, and U373^gH/gL^ cells treated with ZL_3_VS (2.5 µM, 12 hrs) were subjected to immunoblot analysis for gH (lanes 1–6), gL (lanes 7–12), and GAPDH (lanes 13–18). (**D**) U373^gH^, U373^gL^, and U373^gH/gL^ cells were treated with ZL_3_VS as before and subjected to Endoglycosidase H treatment followed by immunoblot analysis. Molecular weight standards and polypeptides are indicated. Arrow represents deglycosylated gH protein.

gH forms a stable disulfide linkage in the ER with the glycoprotein gL^18^,^19^. To investigate whether the increased levels of gH and gL in U373^gH/gL^ cells was due to generation of a gH/gL dimer, we conducted a pulse-chase experiment to analyze the formation of the gH/gL complex (Fig. 3A). U373^gH^, U373^gL^ and U373^gH/gL^ cells were metabolically labeled with ^35^S methionine for 30 minutes and chased up to 60 minutes. gH proteins (Fig. 3A, lanes 1–9), gL proteins (Fig. 3A, lanes 10–27), and properly folded MHC class I molecules (Fig. 3A, lanes 28–36) were recovered from cell lysates and resolved on an SDS-polyacrylamide gel under non-reducing conditions. CMV gH was recovered from U373^gH^ and U373^gH/gL^ lysates throughout the chase period migrating as a ~100 kDa species (Fig. 3A, lanes 1–3 and 7–9), and a protein migrating as a ~25kDa species corresponds to the gL from U373^gH/gL^ cells (Fig. 3A, lanes 22–27). A protein migrating as a ~130 kDa polypeptide recovered exclusively from U373^gH/gL^ cells is the gH/gL covalent heterodimer (Fig. 3A, lanes 7–9 and 16–18) demonstrating that expression of gH with gL forms a covalent dimer. Interestingly, increased levels of gH were recovered from the 30 and 60 minute chase points compared to the 0 min chase point in both U373^gH^ and U373^gH/gL^ cells (Fig. 2A, lanes 1–3 and 7–9). This may be due to the limited exposure of the anti-gH antibody 14-4b epitope early in the chase period.

Consistent with Fig. 2E, the data in Fig. 3A demonstrate that increased levels of gH were recovered from U373^gH/gL^ cells compared to U373^gH^ cells, suggesting that gH is targeted for degradation when expressed alone. To confirm this hypothesis, pulse-chase experiments were carried out to examine the stability of gH in U373^gH^ versus U373^gH/gL^ cells (Fig. 3B). Cells were metabolically labeled for 15 minutes and chased up to 120 minutes. Quantification of radioactivity from the labeled cell lysates demonstrated equal ^35^S methionine incorporation in U373^gH^ and U373^gH/gL^ cells at all chase points (Fig. S2). gH proteins were recovered from cell lysates and resolved on an SDS-polyacrylamide gel under reducing conditions (Fig. 3B, lanes 1–6). As observed in Fig. 2A, low amounts of gH were recovered at the 0 chase point in both cell lines (Fig. 3B, lanes 1 and 4). However, significantly less gH was recovered from the 40 and 120 min chase points of U373^gH^ cell lysates than the same time points for U373^gH/gL^ lysates (Fig. 3B, lanes 2–3 and 5–6). The data indicate that gH molecules are destabilized when expressed alone as compared to gH expressed with gL. As controls, gL complexed with gH was recovered from the U373^gH/gL^ lysates (Fig. 3B, lanes 10–12) and properly folded MHC class I molecules were recovered from all samples (Fig. 3B, lanes 13–18). The results suggest that the stability of gH is enhanced when co-expressed with gL due to the formation of the gH/gL heterodimer.

The increase of gH and gL levels in cells expressing both proteins suggests that the heterodimer may be protected from ER-associated protein degradation (ERAD). To test this, U373^gH^, U373^gL^, and U373^gH/gL^ cells treated with the proteasome inhibitor ZL_3_VS were subjected to immunoblot analysis to analyze levels of gH and gL (Fig. 3C). Proteasome inhibitor treatment of U373^gH^ and U373^gL^ cells resulted in increased levels of both proteins (Fig. 3C, lanes 1–2 and 9–10). Anti-GAPDH immunoblot confirmed equivalent protein loading (Fig. 3C, lanes 13–18). Interestingly, ZL_3_VS treatment of U373^gL^ and U373^gH/gL^ cells revealed a faster-migrating species in addition to the major gL protein that migrates at ~25 kDa (Fig. 3C, lanes 10 and 12, arrow). A band migrating slightly faster than gH was also observed in U373^gH^ cells following proteasome inhibition upon longer film exposure (Fig. 3D, lane 3). The faster migrating species is consistent with a protein that has been dislocated across the ER membrane and into the cytosol where it is deglycosylated by N-glycanase prior to proteasomal degradation^20^. The co-expression of gH and gL likely mitigates this degradation, as no increased gH expression is evident during proteasome inhibition of U373^gH/gL^ cells (Fig. 3C lanes 5 and 6). This was confirmed in a kinetic analysis experiment in which U373^gH^ and U373^gH/gL^ cells were treated with ZL_3_VS for up to 12 hours. U373^gH^ cells demonstrated increased gH expression within 4 hours of treatment while no increase was observed in U373^gH/gL^ cells (Fig. S2). The faster-migrating gL species that appears in U373^gH/gL^ cells following proteasome treatment likely represents monomeric gL that is expressed in excess of gH, and thus does not participate in gH/gL complexes. To confirm that the faster migrating gH proteins were deglycosylated species, cells were treated with ZL_3_VS and half of each cell lysate was exposed to Endoglycosidase H (Endo H), which cleaves high-mannose N-linked glycans of ER resident glycoproteins^21^. Following Endo H treatment, lysates were analyzed by anti-gH immunoblot (Fig. 3D). To permit equivalent exposure of U373^gH^ and U373^gH/gL^ cell lysates, U373^gH/gL^ samples were underloaded compared to U373^gH^ and U373^gL^ samples. As expected, steady-state levels of gH in both U373^gH^ and U373^gH/gL^ cell lysates were sensitive to Endo H cleavage, indicating that the majority of these molecules are trapped within the ER (Fig. 3D, lanes 1–2 and 9–10). Treatment of cells with ZL_3_VS resulted in increased levels of gH in U373^gH^ cells but not U373^gH/gL^ cells, together with the appearance of a faster-migrating gH species in the U373^gH^ cells (Fig. 3D, lane 3). Endo H digestion resulted in collapse of the steady-state gH protein to the faster migrating species induced by ZL_3_VS treatment (Fig. 3C, lane 4), confirming the deglycosylated state of the faster-migrating gH species. Similarly, the steady-state gL protein in U373^gL^ cells treated with Endo H collapsed to the size of the faster species observed following ZL_3_VS treatment (Fig. S2). Together the data support the model that uncomplexed gH and gL molecules are subject to ER quality control and that dimerization rescues the proteins from proteasomal degradation.

### CMV gH/gL dimers traffic to the cell surface

**Figure 4:**
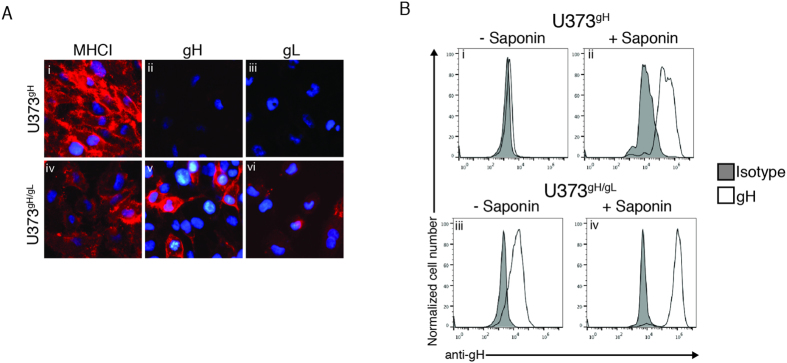
gH/gL dimers traffic to the cell surface. (**A**) U373^gH^ and U373^gH/gL^ cell lines were subjected to analysis by fluorescence microscopy. Cells were probed for properly-folded MHC class I (i and iv), gH (ii and v), or gL (iii and vi) with the respective antibodies followed by incubation with an IgG-specific Alexa-555 fluorescent antibody. Hoechst reagent allowed visualization of the nucleus. (**B**) U373^gH^ and U373^gH/gL^ cell lines were analyzed by flow cytometry for gH expression either without (i and iii), or with (ii and iv) cell permeabilization with saponin treatment. Background fluorescence levels were established by staining with an IgG isotype control.

Previous reports have demonstrated that formation of a complex between CMV gH and gL enable their expression on the cell surface^18^. To confirm these findings and validate the U373^gH^ and U373^gH/gL^ cell lines, we conducted cell-surface immunofluorescence microscopy analysis of U373^gH^ and U373^gH/gL^ cells (Fig. 4A). Detection of properly-folded MHC class I demonstrated appropriate experimental conditions (Fig. 4A, i and iv). CMV gH was not observed on the surface of U373^gH^ cells (Fig. 4A, ii), while U373^gH/gL^ cells displayed cell surface expression of both gH and gL (Fig. 4A, v and vi). To confirm the expression of gH/gL on the cell surface, U373^gH^ and U373^gH/gL^ cells with or without saponin treatment were subjected to flow cytometry. As expected, gH was not observed on the surface of U373^gH^ cells, while permeabilization of the cells with saponin demonstrated detection of intracellular gH protein (Fig. 4B, i and ii). Labeling of U373^gH/gL^ cells demonstrated the surface expression of gH/gL cells as well as increased intensity upon permeabilization (Fig. 4B, (iii and iv)). Isotype controls identified background fluorescence levels in all cells and conditions (Fig. 4B, gray peaks). The data demonstrate that expression of gH alone does not appear sufficient to achieve cell-surface expression in U373 cells; however complementation with gL results in detectable levels of gH at the cell surface.

### The gH transmembrane domain and cytosolic tail influence gH trafficking

**Figure 5:**
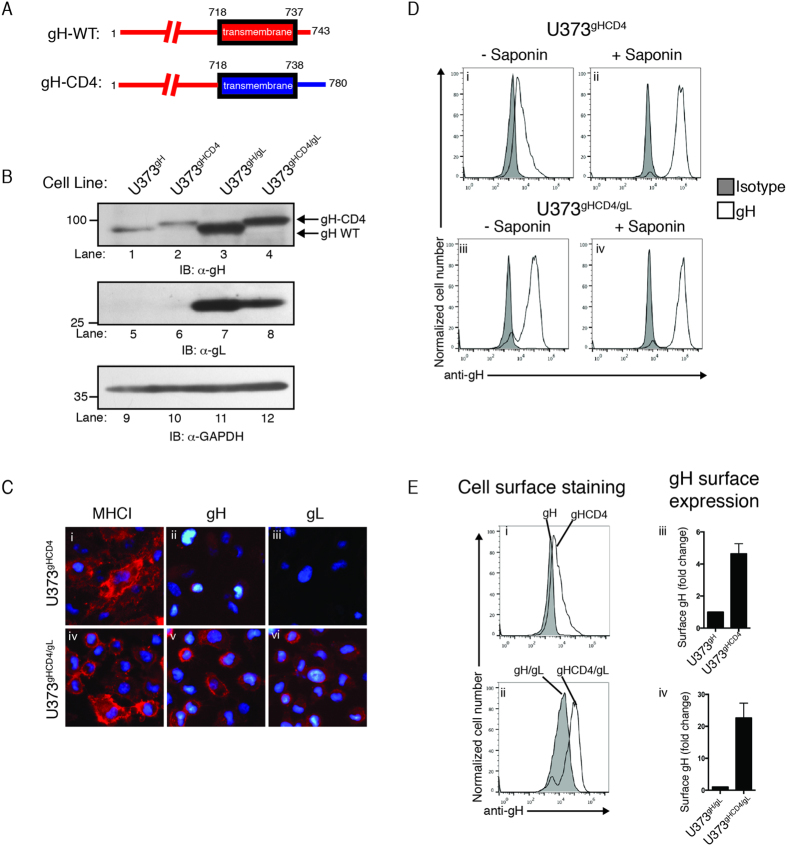
The chimeric gH-CD4 possesses enhanced trafficking properties. (**A**) A schematic representing the luminal and transmembrane domains and the cytoplasmic tail of wild type gH and the gH-CD4 chimeric protein. (**B**) U373^gH^, U373^gHCD4^, U373^gH/gL^, and U373^gHCD4/gL^ cells were subjected to immunoblot analysis for gH (lanes 1–4), gL (lanes 5–8), and GAPDH (lanes 9–12). Molecular weight standards and proteins are indicated. (**C**) U373^gHCD4^ and U373^gHCD4/gL^ cells were subjected to analysis by fluorescence microscopy for properly-folded MHC class I (i and iv), gH (ii and v), or gL (iii and vi) using the respective antibodies followed by incubation with an IgG-specific Alexa-555 fluorescent antibody. Hoechst reagent allowed visualization of the nucleus. (**D**) U373^gHCD4^ and U373^gHCD4/gL^ cell lines were analyzed by flow cytometry for gH expression either without (i and iii), or with (ii and iv) cell permeabilization with saponin treatment. Background fluorescence levels were established by staining with an IgG isotype (gray peaks). (**E**) The amount of cell surface gH versus internalized gH in U373^gH^ and U373^gH/gL^ cells was compared to U373^gHCD4^ (i) and U373^gHCD4/gL^ (ii) cells by flow cytometry. Expression levels were calculated as percent of total gH expressed on the cell surface, and displayed as fold change compared to U373^gH^(iii) and U373^gH/gL^ cells (iv).

The dimerization of gH with gL leads to a properly folded complex that allows the dimer to egress from the ER to the cell surface. Do the membrane anchor and cytoplasmic tail of gH influence its stability or trafficking? We engineered a chimeric gH protein in which the transmembrane and cytosolic domain were replaced with that of the membrane protein CD4 (gH-CD4, Fig. 5A) and generated stable cell lines expressing gH-CD4 (U373^gHCD4^) or gH-CD4 and gL together (U373^gHCD4/gL^). To confirm expression of the chimeric gH-CD4 in the cell lines, U373^gH^, U373^gHCD4^, U373^gH/gL^, and U373^gHCD4/gL^ cells were subjected to immunoblot analysis and gH protein levels were demonstrated in all cell lines (Fig. 5B lanes 1–4). The gH detected in U373^gHCD4^ and U373^gHCD4/gL^ cells migrated slightly slower due to the addition of the larger CD4 transmembrane and cytosolic tail sequence (Fig. 5B, lanes 2 and 4). Anti-GAPDH immunoblot confirmed equivalent protein loading (Fig. 5B, lanes 9–12). Interestingly, the levels of gH-CD4 increased in cells expressing gL suggesting that gH-CD4 is likely subjected to ER quality control and thus gH instability is based on the lumen domain of the gH molecule. We concluded that the gH-CD4 chimera is subjected to similar processing events as the wild type gH.

To determine whether the addition of the CD4 transmembrane and tail would permit the trafficking and cell-surface expression of gH-CD4, cell-surface immunofluorescence analysis was conducted in cells expressing gH-CD4 (Fig. 5C). Detection of properly folded MHC class I demonstrated appropriate experimental conditions (Fig. 5C, i and iv). We observed very faint gH staining on the surface of U373^gHCD4^ cells (Fig. 5C, ii), while U373^gHCD4/gL^ cells displayed robust gH and gL expression on the cell surface (Fig. 5C, v and vi).

To confirm the surface expression of gH-CD4, U373^gHCD4^ and U373^gHCD4/gL^ cells with or without saponin treatment were examined by flow cytometry (Fig. 5D). Strikingly, surface staining of gH-CD4 cells revealed the presence of gH on the cell surface, while cell permeabilization revealed total levels of gH including intracellular gH protein (Fig. 5D, i and ii). Analysis of U373^gHCD4/gL^ cells demonstrated robust surface expression of gH on the cell surface and increased detection of gH upon permeabilization (Fig. 5D, iii and iv). Isotype controls identified background fluorescent levels in all cells and conditions (Fig. 5D, gray peaks). Comparison of the gH surface levels in U373^gH^ versus U373^gHCD4^ cells, and U373^gH/gL^ versus U373^gHCD4/gL^ cells by flow cytometry indicated that the CD4 transmembrane domain enhanced cell surface expression of gH alone or in complex with gL (Fig. 5E, i and ii). The percentage of gH expressed on the cell surface was quantified for all of the U373 cell lines by comparing the mean fluorescent intensity (MFI) of gH in non-permeabilized cells with the MFI of gH in permeabilized cells (Fig. 5E, right column). U373^gH^ cells displayed the lowest levels of cell-surface expression, while U373^gHCD4^ cells expressed an ~5 fold increase in gH surface expression (Fig. 5E, iii). Remarkably, U373^gHCD4/gL^ cells expressed a ~23 fold increase of gH on the cell surface when compared to U373^gH/gL^ cells (Fig. 5E, iv). The data demonstrate that wild type gH requires gL in order to traffic to the cell-surface; however replacement of the transmembrane region and cytosolic tail with that of CD4 leads to a further enhancement of gH cell surface expression.

## Discussion

Through millions of years of coevolution with their host organisms, herpesviruses have developed elegant methods to co-opt cellular processes in order to promote their own agenda. This includes rapid and efficient viral genome replication, alteration of cell death pathways, and immune evasion (Reviewed in^22^). CMV, with its multitude of proteins that target MHC class I for degradation via the ER-associated degradation (ERAD) pathway, offers a quintessential example of the sophisticated ways through which a chronic virus can utilize cellular processes to persist and disseminate^23^.

The data presented in this study support a model wherein the stability and trafficking of the CMV gH protein are modulated by host factors involved in ER-quality control and protein trafficking. gL, a soluble glycoprotein that forms a disulfide bond with gH in the ER lumen, associates with gH following ER translocation. The dimerization of the two proteins permits the proper folding of the gH/gL dimer into a stable complex. Importantly, enhanced gH or gL expression in U373^gH/gL^ cells was not observed following proteasome inhibitor treatment, indicating that the dimerization of gH and gL in the ER protects the complex from proteasome-mediated degradation.

Co-expression of both gH and gL proteins permitted trafficking of the complex to the cell surface (Fig. 4). While gL does not interface with the cytosol, its dimerization with gH may cause critical conformational changes within the complex leading to events that alter the structure of the transmembrane domain or cytosolic tail and permit its exit from the ER. Remarkably, cell-surface expression of gH could be enhanced through replacement of the transmembrane domain and tail with that of the surface glycoprotein CD4 (Fig. 5C,D). Critically, U373^gHCD4^ cells did not display enhanced gH levels when compared to U373^gH^ cells (Fig. 5B). Thus, cell-surface expression of gH-CD4 was not simply the result of increased protein stability. In addition to increasing the amount of gH on the cell surface, co-expression of gL with gH-CD4 further increased total levels of gH-CD4, indicating that the stabilizing effects of gH/gL dimerization may synergize with the enhanced trafficking characteristics of gH-CD4. Overall, the data indicate that the gH transmembrane and cytosolic tail can influence its trafficking and cell surface expression.

Following viral packaging and exit from the nucleus, the nascent CMV virion undergoes various envelopment steps in which it acquires its tegument and full complement of surface proteins. This process is critical, as it will determine the virus’ fitness once it buds from its host cell. Effectively controlled viral glycoprotein folding, complexing, and processing would diminish the possibility of malformed virions and thereby increase overall virus fitness. We propose that CMV utilizes the ERAD pathway to eliminate free gH and gL proteins which do not participate in a heterodimer and could provide a strategy to ensure the proper stoichiometry of gH and gL at the assembly site and the virion surface. A similar model has been described for the ER retention and degradation of the multisubunit complex, T cell antigen receptor (TCR). When the TCRα chain is expressed in the absence of the other subunits of the TCR–CD3 complex, it is dislocated and targeted for proteasomal degradation^24^. Furthermore, as gH is an immunogenic protein that can be potently neutralized by the natural antibody response^25^, limitation of gH exposure on the cell surface would also impart a fitness advantage. Thus, utilization of cellular machinery to sequester gH in the ER until it is released through association with gL would serve to limit the trafficking of gH monomers to the virus assembly site and exposure of the protein to immune surveillance of an infected cell.

The work presented here provides insight into the complex regulation of CMV protein processing in the ER carried out in part through the co-opting of host processes. This study focuses on the specific regulation of gH and gL expression without the full assortment of viral proteins present in the ER during lytic CMV infection. Overall the data highlight the ability of distinct processes to regulate the trafficking of viral glycoproteins, and serve as yet another example of the profound interplay between CMV and its human host.

## Methods

### Cell lines, antibodies, and viruses

Human U373-MG astrocytoma cells and MRC5 lung fibroblasts were cultured in Dulbecco’s modified Eagle’s medium (DMEM) supplemented with 10% fetal bovine serum, 1 mM HEPES, 100 U/ml penicillin, and 100 μg/ml streptomycin at 37 °C in a humidified atmosphere (5% CO_2_). Gpg-293 cells (BD Biosciences) were employed to generate retroviruses and were cultured in identical conditions. The monoclonal anti-gH antibody (clone 14-4b), a kind gift from William J. Britt, was purified from hybridoma culture supernatant^26^. Polyclonal anti-gL immunoglobulins were raised in rabbits by inoculation with a peptide derived from the CMV TB40/E gL sequence (aa. 265–278, PAHSRYGPQAVDAR). Monoclonal antibody W6/32 (anti-MHCI), which recognizes properly folded MHC class I molecules was purified from hybridoma culture supernatant. CMV AD169_IE2-YFP_ was propagated as previously described^16^. Infectious virus yield was assayed on MRC5 fibroblasts by median tissue culture infectious dose (TCID_50_).

### Generation of stable U373 cell lines

gH and gL cDNA from the CMV TB40/E sequence was cloned into the pLGPW and pLHCX vectors (Clontech) and stably introduced into U373 astrocytoma cells by retroviral transduction. gH-CD4 cDNA was generated by replacement of the gH transmembrane and cytosolic tail (aa718–743) with the transmembrane and cytosolic tail of human CD4 (aa397–458). pLGPW stable transfectants were sorted based on GFP expression, and pLHCX stable transfectants were selected in the presence of hygromycin (1.0 µg/mL). U373^gH/gL^ and U373^gHCD4/gL^ cells were first transduced with pLGPW encoding gH or gH-CD4, sorted and then transduced with pLHCX encoding gL.

### Flow cytometry analysis

Flow cytometry analysis was performed as previously described^27^. Briefly, cells were incubated with 1% BSA/PBS, and then anti-gH or anti-gL antibodies followed by secondary staining with Alexa-647 goat anti-mouse or chicken anti-rabbit antibody (ThermoFisher Scientific). Fluorescent data was collected on an Intellicyt HTFC flow cytometer. For analysis of permeabilized cells, cells were fixed with BD Biosciences Cytofix/Cytoperm solution (20 min. at 4 °C) and maintained in 0.1% saponin throughout the staining procedure. The data were quantified by using Flow Jo software (Tree Star, Inc).

### Fluorescent microscopy

U373 cell lines were seeded overnight and then fixed with 4% paraformaldehyde (PFA). Cells were stained using Hoechst reagent and anti-gH anti-gL, or anti-MHC class I antibodies, followed by secondary staining with Alexa-568 goat anti-rabbit or goat anti-mouse antibody (Invitrogen). For analysis of permeabilized cells, cells were maintained in 0.1% saponin throughout the staining procedure. Following wash with PBS, cells were visualized on an EVOS FL microscope.

### Pulse-chase analysis

Pulse-chase experiments with U373 cells were performed as previously described^28^. Cells were pulsed with ^35^S-methionine for the indicated amount of time at 37 °C. Cells were lysed in NP40, pre-cleared with Zysorbin (Invitrogen), and immunoprecipitated for W6/32, gH and gL, followed by incubation with Protein A agarose beads. The recovered protein complexes were then resolved by SDS-PAGE with or without DTT as a reducing agent.

### Analysis of viral protein expression in transgenic cell lines and proteasome inhibition

Following treatment, cells were washed and harvested. Total cell lysates from U373 cell lines were subjected to SDS-PAGE followed by immunoblot analysis using the anti-gH mAb AP86 (gift from William J. Britt)^29^, polyclonal anti-gL IgG, and an anti-glyceraldehyde-3-phosphate dehydrogenase (GAPDH) (Chemicon, Billerica, MA). For proteasome inhibition experiments, cells were treated for 12 hours with the proteasome inhibitor ZL_3_VS (2.5µM). For experiments involving Endoglycosidase H treatment, total cell lysates were incubated with 500 units of Endoglycosidase H_f_ (New England Biolabs) for 2 hours.

## Additional Information

**How to cite this article**: Gardner, T. J. *et al.* Human cytomegalovirus gH stability and trafficking are regulated by ER-associated degradation and transmembrane architecture. *Sci. Rep.* **6**, 23692; doi: 10.1038/srep23692 (2016).

## Supplementary Material

Supplementary Information
