# Supplementary material for: Human cytomegalovirus gH stability and trafficking are regulated by ER-associated degradation and transmembrane architecture
Source: Sci Rep. 2016 Mar 30;6:23692. doi: 10.1038/srep23692 (PMC4812245; doi:10.1038/srep23692)
Supplement: Supplementary Information [file srep23692-s1.pdf]

Human cytomegalovirus gH stability and trafficking are regulated by ER-associated degradation and transmembrane architecture.

Thomas J. Gardner[a], Rosmel E. Hernandez[a], Vanessa M. Noriega[a], and Domenico Tortorella[a,#]

Supplementary Dataset

Supplemental Figure 1

U373 cells

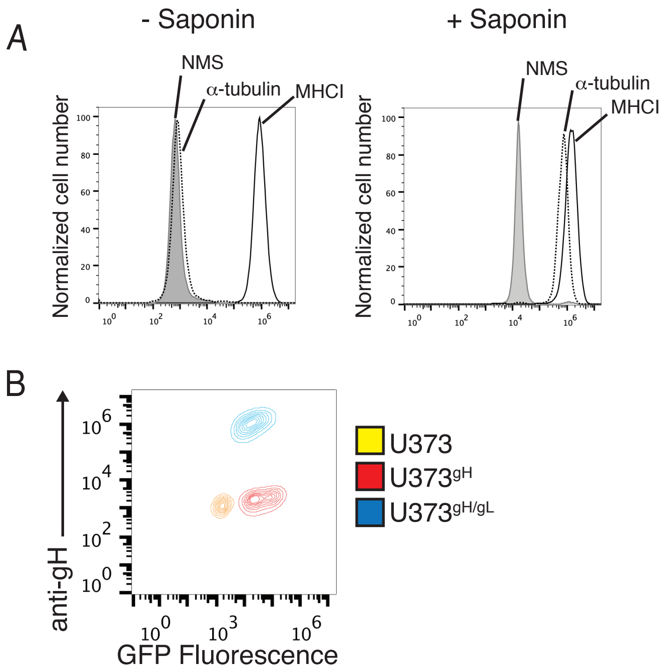

Establishment of flow cytometry conditions for distinguishing between intracellular and membrane surface proteins. (A) U373 cells were analyzed by flow cytometry for α-tubulin and MHC class I expression either with or without cell permeabilization with saponin treatment. Background fluorescence levels were established by staining with normal mouse serum (NMS). (B) U373 (yellow), $U373^{gH}$ (red), and $U373^{gH/gL}$ (blue) cells were analyzed by flow cytometry for gH surface expression. GFP fluorescence (x axis) and gH staining (y axis) were compared by density plot.

Supplemental Figure 2

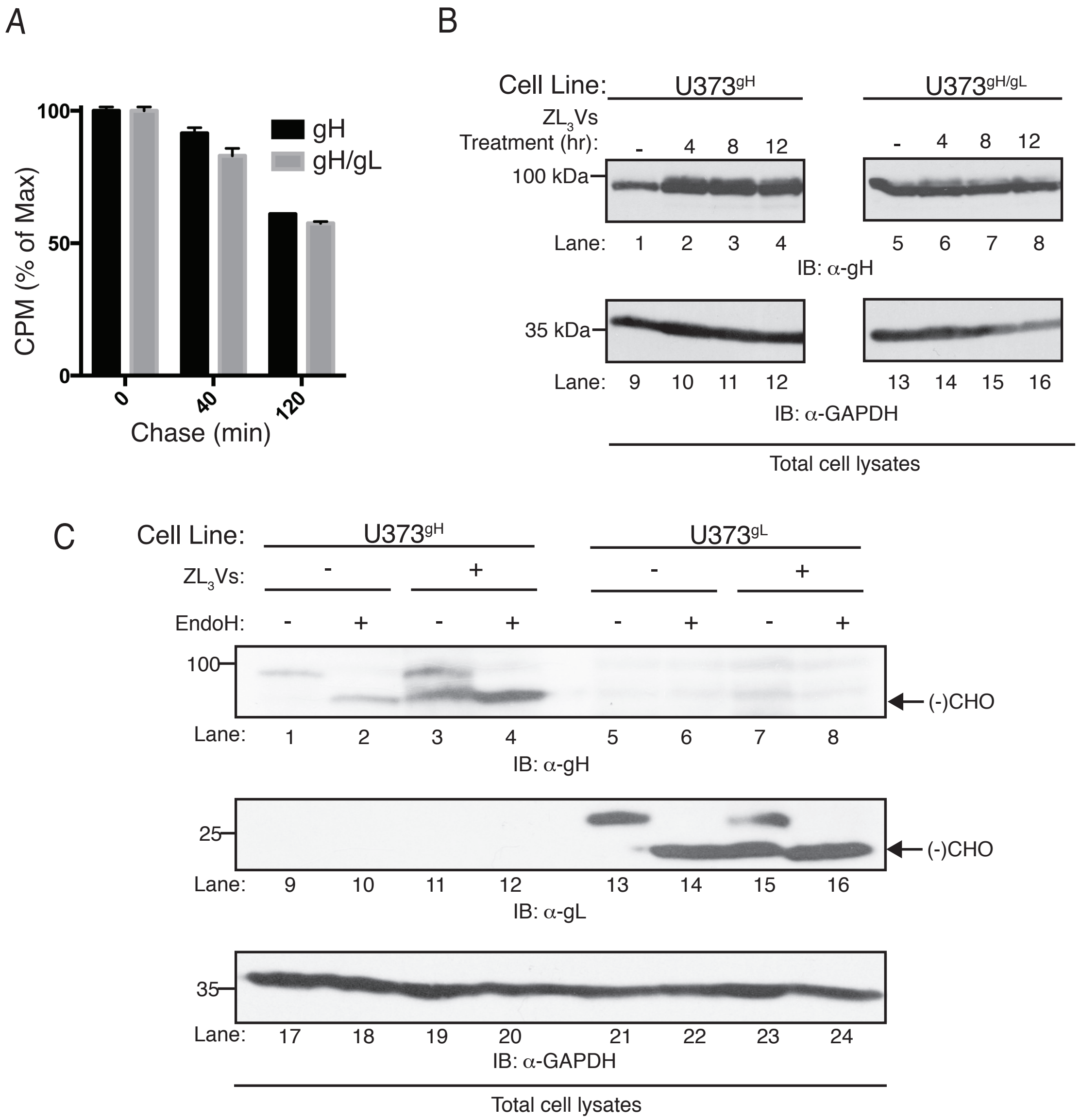

Non-complexed gH and gL are stabilized by proteasome inhibition. (A) Protein from 35S Methionine-labeled cell lysates were precipitated with trichloroacetic acid (TCA) and radioactivity was quantified by liquid scintillation. Values are expressed as percent of maximum counts per minute (CPM) (B) U373^{gH} and U373$^{gH/gL}$ cells were exposed to ZL3VS (2.5 µM) for up to 12 hours. Cells were harvested following 4, 8, and 12 hours of treatment and subjected to immunoblot analysis for gH (lanes 1-8) and GAPDH (lanes 9-16). Molecular weight standards are indicated. (C) U373^{gH} and U373^{gL} cells were treated with ZL3VS (2.5 µM, 12hrs.) and subjected to Endoglycosidase H treatment followed by immunoblot analysis. Molecular weight standards are indicated. Arrows represent deglycosylated gH protein.
